# Double-Blind, Randomized, Placebo-Controlled, Crossover Study of Oral Cannabidiol and Tetrahydrocannabinol for Essential Tremor

**DOI:** 10.5334/tohm.1005

**Published:** 2025-04-14

**Authors:** Katherine Longardner, Qian Shen, Francisco X. Castellanos, Bin Tang, Rhea Gandhi, Brenton A. Wright, Jeremiah D. Momper, Fatta B. Nahab

**Affiliations:** 1 University of California San Diego, Department of Neurosciences, La Jolla, CA, USA; 2 University of California San Diego, Department of Radiology, La Jolla, CA, USA; 3 NYU Grossman School of Medicine, Department of Child and Adolescent Psychiatry, New York, NY, USA; 4 University of California San Diego, Department of Psychiatry, La Jolla, CA, USA; 5 University of California San Diego, Skaggs School of Pharmacy and Pharmaceutical Sciences, La Jolla, CA, USA

**Keywords:** essential tremor, cannabis, therapeutics, digital outcome measures

## Abstract

**Background:**

Essential tremor (ET) is characterized by often disabling action tremors. No pharmacological agent has been developed specifically for symptomatic treatment. Anecdotal reports describe tremor improvement with cannabis, but no evidence exists to support these claims. We conducted a phase Ib/II double-blind, placebo-controlled, crossover pilot trial in participants with ET to investigate tolerability, safety, and efficacy of Tilray TN-CT120 LM, an oral pharmaceutical-grade formulation containing tetrahydrocannabinol (THC) 5 mg and cannabidiol (CBD) 100 mg. Our objectives were to determine if short-term THC/CBD exposure improved tremor amplitude and was tolerated.

**Methods:**

Participants with ET were randomized (1:1) to receive either TN-CT120 LM or placebo. Dose titration, driven by tolerability, was attempted every 2–3 days to three capsules daily maximum. Participants remained on the highest tolerated dose for two weeks before returning to complete assessments. After completing the first arm, participants titrated off the agent, underwent a three-week washout, and then returned for the same procedures with the alternate compound. The primary endpoint was tremor amplitude change from baseline using digital spiral assessment. Secondary endpoints explored safety and tolerability.

**Results:**

Among thirteen participants screened, seven were eligible and enrolled. Five completed all visits; one withdrew following a serious adverse event, and another did not tolerate the lowest dose. Intent-to-treat analyses performed for six participants did not reveal significant effects on primary or secondary endpoints.

**Conclusions:**

This pilot trial did not detect any signals of efficacy of THC/CBD in ET. Although preliminary due to the small sample size, our data do not support anecdotal reports of cannabinoid effectiveness for ET.

**Highlights:**

This double-blind, randomized, placebo-controlled efficacy and tolerability pilot trial did not detect any signals of efficacy of oral cannabidiol and tetrahydrocannabinol in reducing essential tremor amplitude using either digital outcome measures or clinical rating scales. The oral cannabinoids were well-tolerated by most (five out of seven) participants.

## Introduction

Essential tremor (ET) is the most common neurological movement disorder in adulthood, affecting an estimated 1% of the population and about 6% of individuals over age 65 [1]. Essential tremor is defined as bilateral postural and kinetic tremor of the upper extremities that is not attributable to identifiable causes (e.g., stimulant use, medication side effects) and has been present for at least three years [2]. The tremors most commonly affect the hands, head, voice, and legs in order of frequency, leading to impairment in activities of daily living and morbidity. ET often causes social withdrawal, disability, and loss of occupation.

To date, no pharmacological agent has been approved specifically for ET symptomatic treatment, though existing agents such as propranolol and primidone are used to reduce tremor amplitude and improve quality of life [3]. However, these agents frequently cause intolerable side effects and/or provide insufficient benefit [4], so an unmet need exists for better ET treatment. Surgical options such as deep brain stimulation and thalamotomy procedures are reserved for individuals with the most severe tremors that are refractory to medications.

Individuals with ET have long reported tremor benefits from the use of cannabinoids, though only one randomized controlled trial with CBD has been conducted, with negative results [5]. Our pilot study aimed to assess the efficacy and tolerability of TILRAY TN-CT120 LM (Tilray, Canada, Food and Drug Administration (FDA) Investigational New Drug (IND) #137400), a pharmaceutical-grade investigational medicinal product (IMP) formulation containing THC and CBD (5 mg/100 mg per oral capsule, respectively), vs. placebo in ET. Our objectives were to: 1) determine to what extent short-term exposure to oral THC/CBD improved tremor amplitude; 2) determine the acute and short-term tolerability of oral THC/CBD; and 3) characterize the relationship between THC/CBD exposure and tremor amplitude to define a useful dose range for future confirmatory clinical trials.

## Methods

### Standard Protocol Approvals, Registrations, and Patient Consents

The overall goal of this pilot trial (registered at clinicaltrials.gov: NCT03805750) was to determine whether TILRAY TN-CT120 LM is a viable therapy for ET. The study was approved by the Institutional Review Board of the University of California San Diego (UCSD) (Project # 180414) and all study procedures took place at UCSD. Participants were enrolled from January 22, 2019 to February 3, 2020. All participants provided written informed consent during a study screening visit. Since cannabis is a classified as a Schedule 1 agent by the United States Drug Enforcement Agency (DEA), we applied for approval to conduct the trial, along with import/export permits from the DEA and Health Canada, respectively, to receive the investigational medical product from Tilray Canada. Additional review and approval were received from the Research Advisory Panel of California.

### Data Availability

The data that support the findings of this study are available by written request from the corresponding author (KL).

### Screening

Participants with a diagnosis of ET were recruited from the UCSD Movement Disorders Center and the general community. After providing informed consent and being screened for eligibility criteria (see below), participants underwent a complete neurological examination to confirm their diagnosis of ET and exclude other neurological disorders. Cognitive function was measured by the Montreal Cognitive Assessment (MoCA; range 0–30, lower is worse) [6]. Participants who were taking antitremor medications were required to have been on a stable dose of their tremor medications for a minimum of 6 weeks prior to the screening visit and were encouraged to remain on these doses for the remainder of the study period. Participants were asked about their other tremor medication doses at each follow-up visit to confirm stable doses.

### Eligibility Criteria

Inclusion criteria were: 1) ET diagnosed by a movement disorders neurologist (confirmed at screening); 2) age 21 years and older; 3) taking a stable dose of anti-tremor medication (e.g., propranolol, topiramate) for at least 6 weeks prior to screening; and 4) a minimum score of 2 on the Essential Tremor Rating Assessment Scale (TETRAS) [7] upper limb tremor assessment (tremor amplitude of at least 1–3 cm). Exclusion criteria were: 1) clinically relevant non-ET related abnormal findings on neurological exam; 2) Presence of rest tremor; 3) diagnosis of mild cognitive impairment or dementia; 4) currently pregnant or nursing; 5) women of childbearing potential unable or unwilling to use an effective form of contraception during course of the trial; 6) use of medications known to interact with cannabinoid-type agents; 7) history of alcohol use disorder (based on DSM-5 criteria [8]) or substance abuse; 8) exposure in the past 21 days to primidone or within the past seven days to benzodiazepines, ketoconazole, ritonavir, clarithromycin, rifampin, carbamazepine, St. John’s Wort, digoxin, or other agents known to be strong inducers or inhibitors of CYP3 A, CYP2C19, and CYP2C9; 9) unwillingness to abstain from consumption of grapefruit-containing products that are known to strongly inhibit CYP3 A4; 10) taking concomitant medications that are highly protein-bound with a narrow therapeutic index (e.g., warfarin, cyclosporine, and amphotericin B); 11) unwillingness to take a cannabis-derived agent; 12) allergy or sensitivity to sorbitol, xylitol, stevia, other natural sweeteners, or cannabis; 13) active use of cannabis or a cannabis-derived product (within 4 weeks of screening) or unwillingness to abstain from recreational use during the study; 14) history of clinically relevant Axis I psychiatric disorder(s) (e.g., mania, bipolar depressive disorder, schizophrenia, schizoaffective disorder, or other major psychiatric disorder) [8]; 15) active or prior history of suicidal ideation and/or behavior; 16) clinically relevant coagulopathy, immunologic, gastrointestinal, respiratory, cardiovascular (e.g., uncontrolled hypertension, myocardial infarction in the last 18 months, bundle branch block, congestive heart disease), or endocrine (e.g., uncontrolled diabetes mellitus, hyperthyroidism) disorder; 17) current or chronic infection; or 18) renal insufficiency (estimated glomerular filtration rate < 60 mL/min/1.73 m^2^).

### Randomization

After completing the screening visit, participants were assigned in a ratio of 1:1 to two possible sequences (order of the treatment: placebo ➔ THC/CBD and THC/CBD ➔ placebo) based on a pre-set randomization list that was developed by the research pharmacy. THC/CBD and placebo were both provided by the manufacturer in matching capsules, preventing participants from discriminating which agent they were taking. The participants and all study team members were blinded to which agent was administered. Participants were asked which treatment they thought they were receiving at each assessment visit and during the telephone follow-up visits to assess potential unblinding.

### Study Design

**Figure 1:**
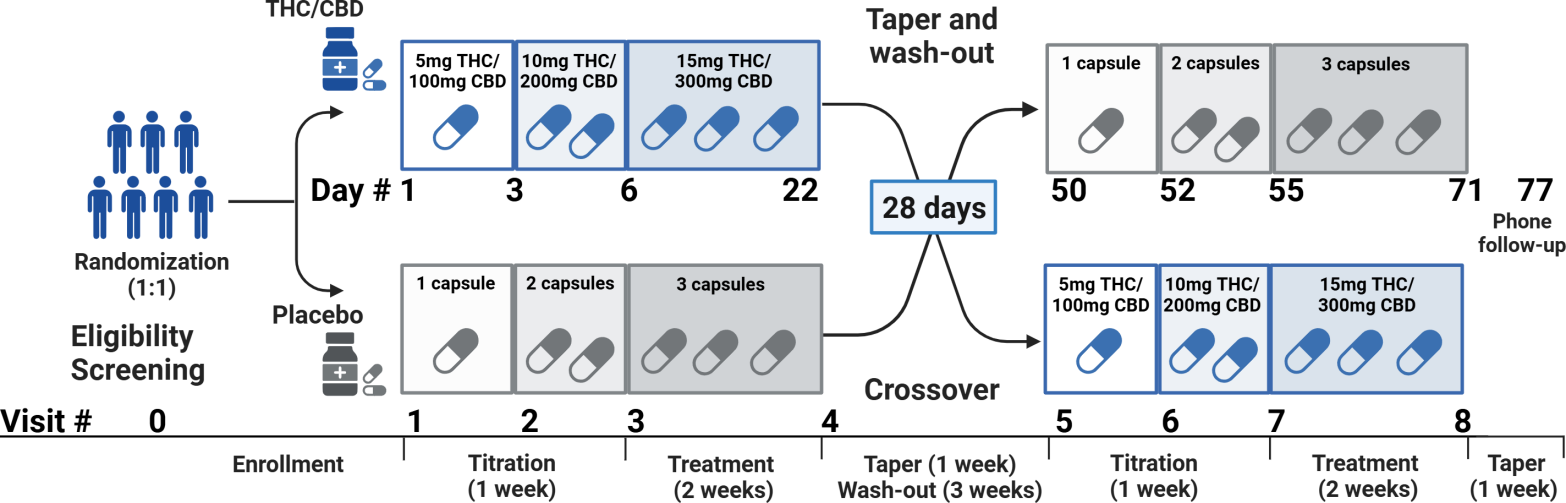
**Schema of the study protocol**. Each participant underwent a baseline assessment and eight follow-up assessments. Each arm of the study period included a one-week titration period, a two-week treatment period, and a one-week tapering period, with a three-week washout period between the two study arms.

This was a phase 1b/II, double-blind, placebo-controlled, crossover trial of THC and CBD for ET. Each participant had a baseline assessment, and eight follow-up assessments as schematized in Figure 1. Each study period included one-week titration, two-week treatment, and one-week tapering. There was a three-week washout period between the two study arms.

### Dose Titration, and Treatment

**Figure 2:**
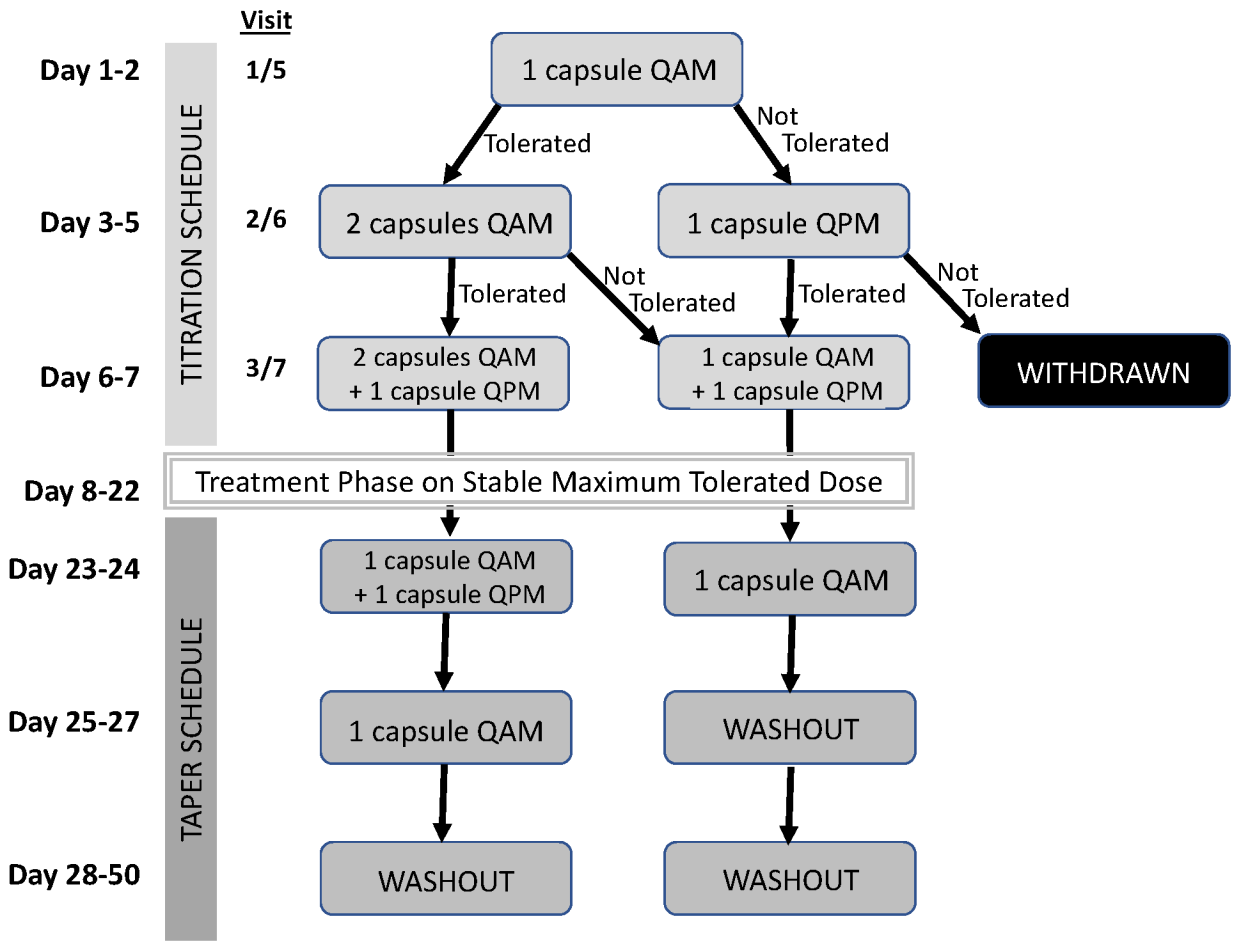
**Titration and taper schedule of the study drug**. The same titration schedule was used for TN-CT120 LM (each capsule contained THC/CBD 5/100 mg) and identical-appearing placebo capsules. Participants remained on the highest tolerated dose throughout the treatment arm.

The study drug dose titration is shown in Figure 2. The same titration schedule was used for TN-CT120 LM and placebo. Participants remained on the highest tolerated dose throughout the treatment arm. The starting dose, titration schedule, and target dose were selected based on safety, tolerability, and efficacy data reported in previous studies using cannabinoids to treat pain [9,10,11]. Adverse effects were inventoried at each study visit. If the participant or investigator felt the higher study drug dose was causing troublesome adverse effects, the dosage could be lowered. During the study design phase, regulators recommended to study cannabis in a ‘real world’ environment, knowing that dietary fat enhances systemic exposure of oral THC and CBD [12,13], and food intake increases the bioavailability of CBD [14]. Participants were advised to schedule their dose at a similar time every morning after breakfast to maintain similar systemic exposure throughout the study. Regular reminders were provided to participants to ensure they were taking the study drug post-meals. During the mornings of the assessment days, participants were provided with a standardized FDA high-fat, high-calorie meal (1000 calories, 50% fat) prior to taking the study drug to evaluate real-world food effects.

### Dose Taper and Washout

After completing a two-week course on the highest tolerated dose of THC/CBD (up to 15/300 mg/day) or placebo, participants remained on this dose and returned for assessment and completion of research measures at visits 4 and 8. Participants were then tapered gradually over one week, similarly to the initial upward dose titration. Participants completed a three-week washout period prior to being crossed over to the alternate arm to allow return to baseline. Washout duration was selected taking the elimination half-life of THC/CBD into consideration [15].

### Assessments

For this digital outcome measure, each participant was asked to trace an Archimedes spiral displayed on a tablet PC screen (IBM Thinkpad X61) twice with each unsupported hand, proceeding from inside the figure to outside. This technique provides an objective measure of tremor and may enable detection of efficacy with fewer participants [16]. Participants with severe tremor who have difficulty even attempting the task are not appropriate for this test, though no such participants were excluded in this study. The tremor amplitude measured by the digital spiral drawing was chosen as the primary outcome. We have previously validated this method using an algorithm to derive the maximum and mean tremor amplitude from the spiral drawings in this cohort with ET [17]. Our prior work demonstrated that this digital measure has a high correlation with manual measures of tremor amplitude and human ratings of the TETRAS score for spiral drawing severity, with excellent test-retest reliability. At each visit, digitized spiral drawings and accelerometry were assessed at baseline (time 0) and at six time points after taking the study drug (15, 25, 60, 100, 200, and 230 minutes).

Tremor severity was rated using the TETRAS [7]. To avoid examiner placebo effects, participant evaluations on this rating were videotaped for subsequent assessments by two blinded raters at the completion of the study. This was a secondary outcome measure. The Global Impression of Change (CGI) scale [18] was used to rate the degree of change from baseline. All adverse events were assessed and recorded using the Patient Reported Outcomes of the Common Terminology Criteria for Adverse Events (PRO-CTCAE) version 1.0 [19]. Suicidality was assessed by trained study personnel using the Columbia-Suicide Severity Rating Scale (C-SSRS) [20]. Blinding assessment was performed at visits 4 and 8; participants and clinicians were asked their opinion of whether participants were in the treatment or placebo arm. Detailed descriptions of the secondary assessments can be found in the Supplementary Appendix.

### Pharmacokinetic Analyses of Cannabinoids in Plasma

Plasma concentrations of cannabinoids (CBD, THC, and their metabolites, including 7-OH-CBD, 11-OH-THC, 7-COOH-CBD, CBG, CBN, THC-COOH, and THC-V), were measured using published methods [21]. Plasma concentrations were obtained pre-dose and 50–240 minutes post-dose at weeks 1 and 2 (visits 2, 3, 4/6, 7, 8) in both the active drug and placebo arms. Plasma concentration-time data of CBD, THC, 7-OH-CBD, 11-OH-THC, 7-COOH-CBD, CBG, CBN, THC-COOH, and THC-V were analyzed by standard non-compartmental methods (Phoenix WinNonlin version 8.4). Maximum and minimum concentrations (C_max_, C_min_) along with corresponding time points (T_max_, T_min_) were observed directly. Area under the concentration time curve (AUC) was estimated by the trapezoidal rule. The correlations between pharmacokinetic (PK) exposures with demographic factors (age, body size) were examined, along with planned analysis of any relationship between exposure and the resulting efficacy and/or safety response.

### Outcome Measures

The primary study outcome was digitized spiral (SPR) amplitude, including maximum, mean, and tremor power features. Maximum amplitude represents the largest deviation from an ideal spiral in millimeters, while mean amplitude represents the average of all tremor amplitudes within a single spiral. Tremor power represents a unitless measure of the intensity of tremor within a peak tremor frequency for a particular spiral. The spiral algorithm calculates rhythmicity by measuring a narrow frequency band at a peak frequency. Secondary outcome measures included the TETRAS [7], CGI [18], PRO-CTCAE [19], plasma concentrations of THC/CBD and their metabolites, and the C-SSRS [20]. Vital signs and ECGs were assessed since the safety of cannabinoids in older adults are not well characterized and cardiovascular effects, including increased heart rate and decreased blood pressure, have been reported in healthy individuals. Serious adverse events such cardiac arrhythmias and acute myocardial infarctions have also been reported [22].

### Sample Size Estimation

A sample size of 14 participants was selected for this exploratory proof-of-concept study as a practical and feasible choice to gather preliminary data and insights, given the absence of prior information in this population to guide a formal power calculation. However, due to multiple constraints (e.g., expiration of IMP, resource/funding limitations) that were exacerbated by the COVID-19 pandemic, we were unable to reach the target sample size.

### Data Analysis

#### Primary analyses

A conservative intent-to-treat approach was used. That is, analyses were performed with all available data (N = 7). Measures of the SPR amplitude (maximum, mean, and tremor power) were summarized with mean (SD) at each time point (0, 15, 25, 60, 100, 200, and 230 minutes) of the fourth visit in each study period (visits 4 and 8) after the two-week treatment by the treatment arm. Since many values were missing at the last two time points (time = 200 and 230 minutes post-dose) due to a change in the study protocol, the treatment efficacy was evaluated by difference (Δ) of the change in SPR measures from baseline to time = 100 minutes post-dose between the two arms using one-sample t-test. The missing Δ values were imputed from the top half (highest 50%) of the observed values [23]. Since the three primary outcomes were related, p-values were corrected for multiple testing using the Benjamini-Hochberg (BH) procedure [24]. In addition, change in measures of amplitude over time and average change from baseline to time = 60, 100, 200, and 230 minutes after dose were compared between placebo and THC/CBD treatments using linear mixed-effects (LME) models with fixed effects of treatment arm, time, and their interaction, and subject-specific random intercepts. Mean response profile was performed, where time was treated as a categorical variable with study baseline and seven time points. In randomized controlled trials, the mean response at baseline is expected to be equal for the two arms; thus, we ran the LME models without a main group effect to increase the power to detect interaction effects. Outcomes were log_10_ transformed prior to analysis to improve normality of data distribution. The effects of the study period and hand side were then estimated and removed from the model if p > 0.2.

The effect of THC/CBD treatment on secondary endpoints were estimated using mixed effects logistic regression, paired samples t-test, linear mixed-effects model, and Wilcoxon signed-rank test (for paired samples). Types of adverse events were summarized with number (%) in aggregated form and by arm.

## Results

### Baseline Characteristics

**Figure 3:**
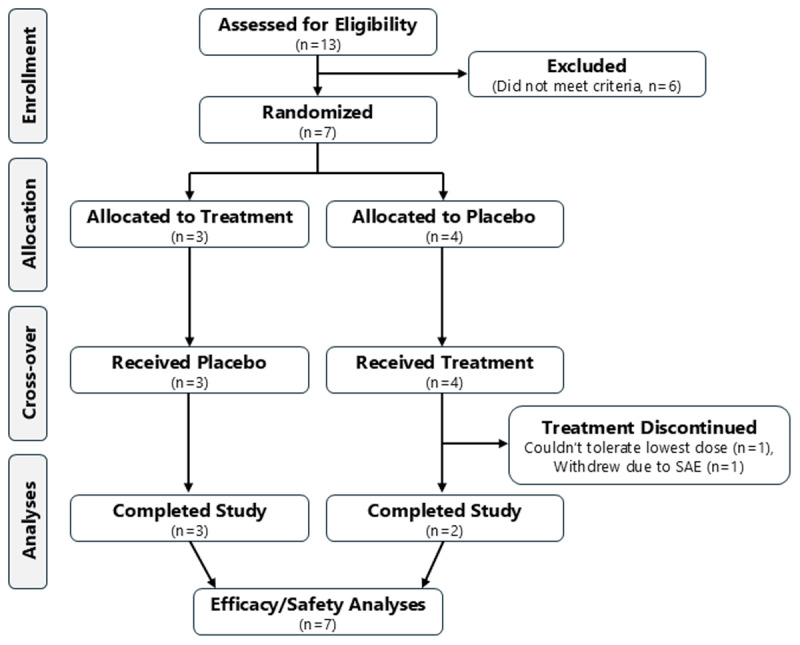
**CONSORT diagram of randomized, placebo-controlled, crossover trial**. Consent was obtained and participants were screened; eligible participants were randomized in a 1:1 ratio to receive either oral THC/CBD 5/100 mg or placebo first.

A total of 13 participants were screened and seven were enrolled; six were ineligible (reasons for exclusion included diagnoses of Parkinson disease (n = 2), primary writing tremor (n = 1), and functional tremor (n = 1)) (Figure 3). Table 1 shows the baseline demographic and clinical characteristics of participants enrolled. Study participants were all White men (100%) in their late 60s (67.0 ± 11.3 year), who had positive family history of ET (100%) and mean ET duration of around 40 years. Only one of the seven participants (14.3%) was taking other medications for tremor at the time of enrollment. This participant, along with four others, had previously tried and failed medications for ET. Participants were asked about prior substance use during screening; four participants reported prior cannabis use years earlier.

**Table 1:** Baseline demographic and clinical characteristics of the randomized participants (N = 7).


Age, years	67.0 (11.3)

Sex, male	7 (100%)

Race	

White	7 (100%)

Ethnicity,	

Non-Hispanic/Latino	7 (100%)

BMI	27.3 (4.7)

Duration of ET, years	39.5 (19.4)

Age at tremor onset, years	26.7 (20.3)

Positive family history of ET	7 (100%)

Alcohol responsive tremors	6 (85.7)

MoCA	27.4 (1.9)

Currently taking other medications for tremor	1 (14.3%)

Previously tried and failed medications for tremor	5 (71.4%)

TETRAS Part 1	28.3 (5.0)

TETRAS Part 2	26.9 (3.2)

Baseline tremor amplitude on digital spiral drawing, mm	

More affected hand, maximum	9.4 (4.8)

More affected hand, mean	2.9 (1.2)

Less affected hand, maximum	4.9 (2.7)

Less affected hand, mean	1.48 (0.4)


Continuous variables are reported as mean (SD); categorical variables are reported as N (%). Abbreviations: BMI = Body Mass Index; ET = essential tremor; mm = millimeters; MoCA = Montreal Cognitive Assessment; TETRAS = The Essential Tremor Rating Scale.

### Treatment effects

Seven participants were randomized (N = 7; four in the placebo to THC/CBD sequence and three in the THC/CBD to placebo sequence). Two participants (both in the placebo to THC/CBD sequence) withdrew from the study during the second period while taking the study drug; one had a serious adverse event, and the other was unable to tolerate the lowest dose of one capsule. The mean (SD) study baseline SPR measures (maximum, mean, and tremor power) were 7.16 mm (4.40), 2.18 mm (1.17), and 0.051 (mm/s)^2^/Hz, respectively.

### Primary Analysis

**Figure 4:**
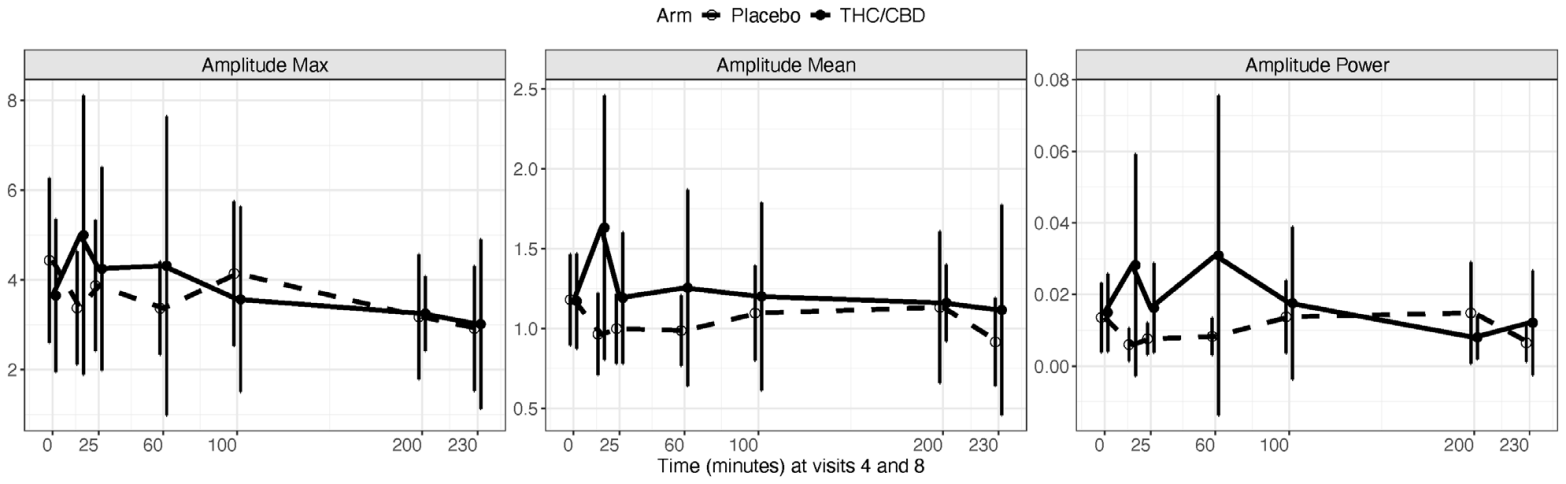
**Tremor amplitude measured by digital spiral drawing over time**. The digital spiral drawings were repeated at various time points ranging from time = 0 [pre-dose] to 230 minutes after dose at visits 4 and 8 at the end of the two-week treatment period in the THC/CBD and placebo arms. Error bars indicate 95% confidence intervals.

We found no evidence to support greater tremor reduction in the THC/CBD arm compared to placebo (maximum amplitude: mean difference Δ = –1.02, effect size *d* = –0.43, *p* = 0.13; mean amplitude: Δ = 0.083, *d* = 0.025, *p* = 0.37; amplitude power: Δ = 0.0004, *d* = –0.026), *p* = 0.92) (Table 2). Also, average change from baseline to time = 60, 100, 200, and 230 minutes post-dose was not significantly different between the arms (maximum amplitude: *χ*^2^ = 2.81, df = 1, adjusted *p* = 0.15; mean amplitude: *χ*^2^ = 0.77, df = 1, adjusted *p* = 0.38; amplitude power: *χ*^2^ = 2.71, df = 1, adjusted *p* = 0.15) (Table 3). In addition, changes in the primary outcomes over time (from baseline to 230 minutes after dose) did not significantly differ between placebo and THC/CBD treatments (Table 3). Figure 4 depicts mean amplitude measures with 95% confidence interval (CI) for each time point.

**Table 2:** Comparisons of changes in primary outcomes from baseline to time = 100 minutes after dose between treatment arms at visits 4 and 8 at the end of the two-week treatment period for all enrolled participants using paired t-test.


SPR	PLACEBO ARM T = 100 MEAN (SD)	THC/CBD ARM T = 100 MEAN (SD)	DIFFERENCE^a^ (95% CI)	COHEN’S D (95% CI)	P-VALUE	ADJUSTED P-VALUE^b^

Amplitude, max (mm)	4.14 (3.07)	3.57 (2.98)	–1.02 (–2.41, 0.36)	–0.43 (–1.01, 0.15)	0.13	0.40

Amplitude, mean (mm)	1.10 (0.57)	1.20 (0.85)	–0.083 (–0.27, 0.11)	–0.25 (–0.83, 0.33)	0.37	0.73

Power (mm/s)^2^/Hz	0.014 (0.019)	0.018 (0.031)	0.0004 (–0.0095, 0.0087)	–0.026 (–0.60, 0.55)	0.92	0.92


Note: ^a^Difference of change in SPR measures from baseline to time = 100 between the two arms. ^b^p-values were adjusted for multiple related outcomes using the Benjamini-Hochberg method; Abbreviations: SPR = digital spirography; CI = confidence interval.

**Table 3:** Comparisons of change over time and average change in mean (standard deviation) of digital spiral drawing measures between THC/CBD and placebo arms at each time point after two-week treatment at visits 4 and 8 using linear mixed-effects models by treatment arm.


	OUTCOME^a^	*χ*^2^ (DF)	P-VALUE^b^	ADJUSTED P-VALUE^c^

Change over time (from baseline to time = 230 minutes after dose)	SPR (Amplitude, max) (mm)	9.64 (7)	0.21	0.63

	SPR (Amplitude, mean) (mm)	3.69 (7)	0.81	0.81

	SPR Power (mm/s)^2^/Hz	5.70 (7)	0.57	0.81

Average change from baseline to the last four time points (60 100, 200, 230 minutes after dose)	SPR (Amplitude, max) (mm)	2.81 (1)	0.093	0.15

	SPR (Amplitude, mean) (mm)	0.77 (1)	0.38	0.38

	SPR Power (mm/s)^2^/Hz	2.71 (1)	0.10	0.15


Note: ^a^log_10_ transformation prior to analysis; ^b^p-value for interaction effect; ^c^p-values were adjusted for multiple related outcomes using the Benjamini-Hochberg method; Abbreviations: SPR = digital spirography; df = degrees of freedom.

Similar results were obtained for analyses stratified by right and left hands, dominant and non-dominant hands, and worse and not-worse hands (data not shown). Results showed that all treatment arm by time interaction effects were not significant, which indicates that the mean response profiles from the time zero (pre-dose) to 230 minutes after dose did not differ between placebo and THC/CBD treatments.

### Secondary Endpoints

#### TETRAS

Change in TETRAS total score from pretreatment baseline (visit 0) to the end of each treatment week (visits 4 and 8) was compared between the treatment (–10.30 ± 3.90) and placebo (–8.07 ± 5.71) arms using a mixed-effects model with participant-specific random intercept. The changes did not differ between placebo and THC/CBD treatment arms (*p* = 0.27).

#### CGI-PGI

CGI and PGI were compared between the two treatment arms using Wilcoxon signed-rank test. Neither CGI nor PGI differed between placebo and THC/CBD treatment arms (*p* = 0.35).

#### Montreal Cognitive Assessment (MoCA)

The MoCA total score did not differ between the two treatment arms (*p* = 0.47).

#### Vital Signs and ECG

We evaluated changes in both seated blood pressures and orthostatic blood pressures by repeating the blood pressure assessment at three minutes post-standing. Compared to the placebo arm, the three-minute standing diastolic blood pressure was lower (B [95% CI] = –4.81 [–7.57, –2.06], *p* = 0.001) and sitting diastolic blood pressure and sitting pulse were marginally lower (–1.91 [–4.10, 0.28], *p* = 0.09; –1.68 [–3.56, 0.21], *p* = 0.085, respectively) in THC/CBD arm. The three-minute standing and sitting systolic the blood pressure and three-minute standing pulse rate did not differ significantly between the two treatment arms. Temperature and respiratory rate did not differ between the two treatment arms (*p* = 0.61 and *p* = 0.49, respectively). ECG measures assessed included heart rate, PR interval, QRS duration, and QTc interval. We explored changes in ECG from baseline at each study visit for treatment and placebo arms. Mixed-effects models showed that changes of any ECG indices were not significantly different between the treatment arms.

### Blinding Assessment

Both the clinicians and the participants were 91.7% accurate in correctly determining the treatment arm.

### Adverse Events

Seven enrolled participants reported a total of 101 adverse events over the course of the study. Among these, 28 (29.8%) were reported during the placebo condition, 66 (70.2%) were reported during the THC/CBD condition, and seven were reported during the taper period (Supplementary Table 1). There were no Adverse Effects of Special Interest.

One serious adverse event (SAE) was recorded. On the day of the event, the participant boarded a flight at 6am and took the prescribed three capsules of IMP containing THC/CBD at 6:30am but had not eaten as required by the protocol. At 7am, the participant ate breakfast and then began to “feel high” and lightheaded, while his spouse noted he appeared pale. At 8:30am, the participant went to the bathroom on the plane and within minutes of returning to his seat (~8:45am) his spouse described a spell consisting of his head and eyes rolling back, then tonic-clonic posturing that lasted approximately 1–2 minutes, followed by emesis. The participant had no prior history of seizure. Medical personnel on the flight assessed vital signs that were reportedly normal though no documentation was available. The participant remained pale and felt exhausted for a period of 20 minutes before returning to baseline. Upon flight landing, he was taken to a local emergency department for additional evaluation. Upon arrival, blood pressure readings could not be obtained due to hypotension. Laboratory and imaging studies were normal with bradycardia (heart rate 43 beats per minute) noted on ECG. The participant received intravenous fluids and was discharged hours later with a diagnosis of syncope. He had no further sequelae, though he discontinued further use of the study drug and was withdrawn from the study. The event was also reviewed by the study data safety monitoring board, which concurred with the suspected diagnosis of convulsive syncope that had a reasonable possibility of being study related.

### Pharmacokinetic Results

The pharmacokinetic results from visits 2, 3, 4, 6, 7, and 8 are shown in Supplementary Table 2 for six participants; data was missing for participant P02, whose samples were collected but were not found during processing. The median Tmax for THC and CBD was 4.0 hours (range = 1.5–4.0 hours) and 3.0 hours (range: 1.5–4.0 hours), respectively. The mean adjusted Cmax for CBD was 0.31 ng/mL/mg (SD = 0.24) and for THC was 0.32 ng/mL/mg (SD = 0.26). Concentrations of CBD, THC, and their metabolites varied among and within individuals, with only one participant, P07, showing a consistent concentration of the predominant metabolite, 7-COOH-CBD. Several metabolites, including CBN, CBG, and THC-V, were below the limit of quantitation in plasma. While we observed a consistent washout in THC, low concentrations of CBD and its metabolites (7-OH-CBD and 7-COOH-CBD) were apparent for some participants in the drug –> placebo arm, with 7-OH-CBD detectable in some participants through visit 8, although there was a ~98% reduction in levels compared to the drug arm.

## Discussion

This pilot study of a pharmaceutical-grade formulation containing THC and CBD (TN-CT120 LM), was conducted to determine if short-term exposure to orally administered THC/CBD reduced tremor amplitude in ET and to determine acute and short-term safety and tolerability of orally administered THC/CBD. The study intended to enroll 14 participants to provide sufficient statistical power given prior expectations. A total of 13 participants were screened, but only seven met inclusion/exclusion criteria, and all were enrolled. A key goal of this trial was to determine tolerability of orally administered cannabis in this study population (mean age: 67.0 ± 11.3 years). We found varying degrees of tolerability that differed by dose and by individual. Some THC/CBD was tolerated by most (5 of 7 participants), though only a single participant tolerated the highest dose and could not tolerate the three capsules all administered together (instead, the tolerable regimen was two capsules every morning and one capsule nightly), while one individual could not tolerate the lowest dose of the active compound, which contained 5 mg of THC and 100 mg of CBD, due to “feeling altered”. All seven participants were included in the intent-to-treat analyses. The study drug may not have been as well-tolerated in our cohort as in previous studies using cannabis for treatment of chronic pain [25] due to factors such as our older study population, as well as differences in dosing and formulation. We evaluated several safety measures and found a favorable safety profile, with the only notable cardiovascular effect being a diastolic orthostatic blood pressure drop of 4.8 mmHg after three minutes of standing (*p* = 0.001), which could potentially contribute to symptoms of lightheadedness.

Our study provides novel pharmacokinetic results about cannabinoids from a population of older adults that were not habitual cannabis users. Although interpretation of our data is limited by the small sample size, our findings for time to peak plasma concentrations for THC and CBD are within the ranges previously reported for pharmaceutical-grade oral cannabinoid formulations, which span between 1–6 hours [26]. Notably, oral cannabinoids can have unpredictable absorption, with low bioavailability (about 6% [27]) due to first-pass liver metabolism and high intra- and inter-individual variability in pharmacokinetic parameters [28]. This difference may also be influenced by dietary factors – in our study, all participants ate a standardized high-calorie, high-fat meal prior to taking the study drug. In comparison, inhaled cannabis formulations have a much shorter time to peak plasma concentrations (estimated 3–10 minutes) and higher bioavailability, estimated at 10–35%, depending on the frequency of use and inhalation duration and depth. Of note, the only SAE occurred in the context of fasted intake, suggesting that the fed-state carried out during this study may have contributed to slowed gastric emptying and a slower time to peak absorption that potentially improved tolerability. While bioavailability (AUC) may be higher with food [13,14], the SAE suggests that a shorter Tmax and a higher Cmax may have been contributory, although we have no additional data to justify this.

Despite the limitations in our sample size and homogeneous demographics of participants (all were White men), we found no evidence to support the hypothesis that cannabis reduces the amplitude of essential tremors in the wide dose range we studied (THC 5–15 mg and CBD 100–300 mg/day). There is no data to suggest that lower doses would be more beneficial. A review of CBD in clinical populations suggested overall better clinical effects with higher dosing [15]. A recent review of clinical trials using THC for other indications reported that doses comparable to those used in our study improved pain and social stress [29]. We acknowledge that it remains uncertain whether a longer treatment duration may have led to further tremor reduction. However, in the subacute exposure phase examined in this study, we saw no trend toward tremor improvement, with PK data remaining relatively stable across assessments in the treatment arm. Given the PK results showing low concentrations of CBD metabolites persisting through week 7 of the placebo arm in the treatment to placebo group, we would recommend a longer washout period between the arms in future studies. The previously reported washout period in the literature for cannabis was up to 14 days [13,30]. Our data suggest that in crossover studies using higher doses of CBD, the washout period reported in the literature is inadequate. Participants in both groups had substantial decreases in ET measures from baseline to the blinded treatment phases, with the maximum spiral amplitude at the last timepoint of the week 3 visits improving in both arms by about 50% from the baseline visit. Potential explanations for similar improvements in both treatment and placebo arms may reflect a combination of factors including initial anxiety amplifying the tremor that subsides over time along with learning effects. A previous study suggested that in patients with ET, the reduction in tremor severity during repeated spiral drawings plateaus after the fourth repetition [31]. There were no statistically significant differences between treatment and placebo arms. We defined the minimum clinically meaningful effect size in this study as a 30% improvement compared to placebo (over baseline). Using a 95% CI of effect size (Table 2) to assess the possibility of inadequate power, the differences in SPR mean and SPR power were outside of the 95% CI, indicating that the study drug is unlikely to have a significant improvement at 100 minutes after dose. The difference for SPR max was inconclusive. Therefore, this is a null study.

To our knowledge, this is the first randomized, placebo-controlled study evaluating safety and efficacy of THC/CBD in ET. Only one other randomized placebo-controlled trial has evaluated cannabinoids in ET – a study of 19 participants with ET evaluated the effect of a single dose of oral CBD 300 mg on tremor amplitude and found no effect on upper limb tremor [5]. There are several challenges to conducting clinical research with cannabis use. In the United States, federally, THC remains classified as a DEA Schedule 1 controlled substance (high potential for abuse) and is highly regulated. Thus, to conduct clinical trials with cannabinoids, it is necessary to work with multiple regulatory agencies. Further, while taking cannabis, participants are not permitted to drive, which complicates the logistics of transportation to and from study visits. Additionally, our study illustrates the challenge of performing a blinded clinical trial with a psychoactive agent, as demonstrated by the very high accuracy rate (~92%) of correctly guessing the treatment arm by both participants and clinicians.

There are numerous reasons for failure in a clinical trial, including selection of inappropriate dosage, poor bioavailability, or non-adherence by participants. In this study, we were able to test a wide dose range, with lower doses being unlikely to have a clinical impact while higher doses would further limit tolerability that we observed. Concerns related to bioavailability were also addressed by pharmacokinetic sampling that we performed throughout the study days, which confirmed the absorption of IMP had occurred at expected levels and negated any possibility of participant non-adherence beyond the routine pill-counting that was also performed at the start of each visit.

While larger trials will continue to test cannabis for various neurological indications, we also identified the need for regulatory and legal reforms to reduce the complexity and burden of conducting rigorous trials. In this study the additional constraints included the expiration of IMP (twice), complexity of US/Canadian regulatory approvals for import/export of new IMP during the COVID-19 pandemic, and resource/funding limitations. Each challenge is surmountable, but the combination limits and slows the development of new findings at a time of limited knowledge and great public enthusiasm. Despite these challenges, our findings reported here provide multiple new key learnings to support the development of future cannabis trials for various neurological disorders.

As the study did not reach its accrual goal of 14 completers, only tentative conclusions can be offered. Oral THC/CBD was tolerated by most participants, although a minority was unable to continue treatment. Rigorous blinded objective and clinical measures did not detect any signals of efficacy of daily doses of up to 15 mg THC and 300 mg CBD in ET.

## Additional Files

The additional files for this article can be found as follows:

10.5334/tohm.1005.s1 Supplementary Appendix. Descriptions of the secondary assessments.

10.5334/tohm.1005.s2 Supplementary Table 1. Description of adverse events by treatment arm.

10.5334/tohm.1005.s3 Supplementary Table 2. Pharmacokinetic results of plasma cannabinoids during the treatment arms collected at visits 2, 3, 4, 6, 7, and 8.
