# Supplementary material for: Double-Blind, Randomized, Placebo-Controlled, Crossover Study of Oral Cannabidiol and Tetrahydrocannabinol for Essential Tremor
Source: Tremor Other Hyperkinet Mov (N Y). 2025 Apr 14;15:14. doi: 10.5334/tohm.1005 (PMC12005140; doi:10.5334/tohm.1005)
Supplement: Supplementary Appendix. — Descriptions of the secondary assessments. [file tohm-15-1-1005-s1.pdf]

Supplemental File 1: Descriptions of secondary assessements

The TETRAS is an examiner-administered scale that evaluates tremor of various body parts during posture, kinesis, and tasks. Items are scored from 0 to 4, with 4 representing the highest degree of tremor severity, and the maximum score is 64[6]. To avoid examiner placebo effects, participant evaluations on this rating were videotaped for subsequent assessments by two blinded raters at the completion of the study. This was a secondary outcome measure.

The Global Impression of Change scale[15] is a standardized and commonly used 7-point scale in which both the participant (PGI) and blinded clinician (CGI) rate the degree of change from baseline.

All adverse events were assessed and recorded using the Patient Reported Outcomes of the Common Terminology Criteria for Adverse Events (PRO-CTCAE) version 1.0[16]. This standardized tool was used to identify and rate severity of adverse events across all organ systems.

Suicidality was assessed by trained study personnel using the Columbia-Suicide Severity Rating Scale (C-SSRS)[17]. The interview measures presence of suicidality and consists of four categories: suicidal ideation, intensity of ideation, suicidal behavior, and answer for actual attempts only. This scale was used for screening as well as to assess for the occurrence of any suicidal ideation and/or behavior during the study.
