# Supplementary material for: Double-Blind, Randomized, Placebo-Controlled, Crossover Study of Oral Cannabidiol and Tetrahydrocannabinol for Essential Tremor
Source: Tremor Other Hyperkinet Mov (N Y). 2025 Apr 14;15:14. doi: 10.5334/tohm.1005 (PMC12005140; doi:10.5334/tohm.1005)
Supplement: Supplementary Table 1. — Description of adverse events by treatment arm. [file tohm-15-1-1005-s2.pdf]

**Supplemental Table 1:** Adverse events by arm, and type of event: N (%).			
Adverse Events	*Placebo arm*	*THC/CBD arm*	*Both arms*
Anxiety	0	2	2 (3%)
Blurred vision	0 (0%)	1 (1.5%)	1 (1.5%)
"Coming down from high"	0 (0%)	1 (1.5%)	1 (1.5%)
Decreased concentration	0 (0%)	7 (10.6%)	7 (10.6%)
Decreased libido	0 (0%)	1 (1.5%)	1 (1.5%)
Diarrhea	0 (0%)	1 (1.5%)	1 (1.5%)
Dizziness	0 (0%)	4 (6.1%)	4 (6.1%)
Dry mouth	0 (0%)	1 (1.5%)	1 (1.5%)
Dyspnea on exertion	4 (14.3%)	4 (6.1%)	8 (20.3%)
Euphoria	0 (0%)	1 (1.5%)	1 (1.5%)
Fatigue	0 (0%)	1 (1.5%)	1 (1.5%)
Feeling relaxed	1 (3.6%)	2 (3%)	3 (6.6%)
Felt "high, deep in thought, with slowed thinking	0 (0%)	1 (1.5%)	1 (1.5%)
Felt “a hit/buzz”	0 (0%)	1 (1.5%)	1 (1.5%)
Felt "stoned"	0 (0%)	1 (1.5%)	1 (1.5%)
Groggy	1 (3.6%)	0 (0%)	1 (3.6%)
Headache	0 (0%)	2 (3%)	2 (3%)
Imbalance	0 (0%)	3 (4.5%)	3 (4.5%)
Improved tremor	0 (0%)	1 (1.5%)	1 (1.5%)
Increased thirst	0 (0%)	1 (1.5%)	1 (1.5%)
Insomnia	2 (7.1%)	0 (0%)	2 (7.1%)
Lightheaded	0 (0%)	2 (3%)	2 (3%)
Memory problems	0 (0%)	3 (4.5%)	3 (4.5%)
Mild headache	1 (3.6%)	0 (0%)	1 (3.6%)
Numbness/tingling	4 (14.3%)	4 (6.1%)	8 (20.3%)
Quivering voice	0 (0%)	1 (1.5%)	1 (1.5%)
Ringing in ears	6 (21.4%)	7 (10.6%)	13 (32%)
Runny nose	2 (7.1%)	3 (4.5%)	5 (11.7%)
Sleepiness	2 (7.1%)	6 (9.1%)	8 (20.3%)
Thumb pain due to sprain	1 (3.6%)	0 (0%)	1 (3.6%)

Tired	1 (3.6%)	0 (0%)	1 (3.6%)
Visual slowing	0 (0%)	1 (1.5%)	1 (1.5%)
Watery eyes	3 (10.7%)	3 (4.5%)	6 (15.3%)
**Total**	28	66	94 (100%)

Notes: The percentage of each adverse event (AE) was calculated by dividing the total count of the corresponding column; the percentage in Total (the last row) represents the proportion of the AE count in placebo arm, THC/CBD arm, or both arms vs. the total AE count.
