# Supplementary material for: Double-Blind, Randomized, Placebo-Controlled, Crossover Study of Oral Cannabidiol and Tetrahydrocannabinol for Essential Tremor
Source: Tremor Other Hyperkinet Mov (N Y). 2025 Apr 14;15:14. doi: 10.5334/tohm.1005 (PMC12005140; doi:10.5334/tohm.1005)
Supplement: Supplementary Table 2. — Pharmacokinetic results of plasma cannabinoids during the treatment arms collected at visits 2, 3, 4, 6, 7, and 8. [file tohm-15-1-1005-s3.pdf]

Supplementary Table 2

Treatment Arm						Placebo Arm
**Analyte**	**T_{max} (hours)**	**C_{max} (adjusted) ng/mL/mg**	**AUC_{0-180} (adjusted) ng*hr/mL/mg**	**AUC_{0-last} (adjusted) ng*hr/mL/mg**	**Mean (SD) across study visits**	**Mean (SD) across study visits**
7-COOH-CBD	1.9 (3.2)	4.61 (2.82)	805.5 (542.7)	1,053.9 (711.9)	758.8 (574.3)	17.19 (53.25)
THC-COOH	4.0 (1.5-4.0)	5.81 (2.38)	746.9 (378.6)	1,005.6 (533.5)	39.5 (32.6)	1.49 (5.52)
CBD	4.0 (1.5-4.0)	0.31 (0.24)	29.5 (17.6)	41.9 (20.3)	25.9 (38.5)	1.19 (2.33)
11-OH-THC	4.0 (1.5-4.0)	0.38 (0.30)	24.3 (29.6)	33.3 (25.1)	1.2 (2.1)	0 (0)
THC	4.0 (1.5-4.0)	0.32 (0.26)	22.1 (20.4)	30.7 (22.5)	0.9 (1.9)	0 (0)
7-OH-CBD	4.0 (1.5-4.0)	0.08 (0.04)	10.2 (5.0)	14.2 (7.5)	10.5 (7.9)	0.38 (1.39)
THC-V	Below limit of quantitation					
CBG	Below limit of quantitation					
CBN	Below limit of quantitation					

Note: Tmax values are reported as median (range). Other values are reported as mean (SD). C_{max} and AUC data are dose adjusted on a per-mg basis for each participant for the relevant study drug (e.g., THC-COOH values are adjusted by the THC dose, while 7-OH-CBD values are adjusted by the CBD dose).
Mean and SD values for the treatment arm and placebo arm are averaged across the three study visits (e.g., visits 2-4 or 6-8) for each arm.

Abbreviations: AUC= area under the plasma concentration vs. time curve to either 180 minutes or the last measured concentration; C_{max}= maximum observed concentration; T_{max}= time of maximum concentration.
